# Deep Learning in Virtual Screening: Recent Applications and Developments

**DOI:** 10.3390/ijms22094435

**Published:** 2021-04-23

**Authors:** Talia B. Kimber, Yonghui Chen, Andrea Volkamer

**Affiliations:** In Silico Toxicology and Structural Bioinformatics, Institute of Physiology, Charité-Universitätsmedizin Berlin, Charitéplatz 1, 10117 Berlin, Germany; talia.kimber@charite.de (T.B.K.); yonghui.chen@charite.de (Y.C.)

**Keywords:** virtual screening, drug-target interaction, deep learning, protein encoding, ligand encoding

## Abstract

Drug discovery is a cost and time-intensive process that is often assisted by computational methods, such as virtual screening, to speed up and guide the design of new compounds. For many years, machine learning methods have been successfully applied in the context of computer-aided drug discovery. Recently, thanks to the rise of novel technologies as well as the increasing amount of available chemical and bioactivity data, deep learning has gained a tremendous impact in rational active compound discovery. Herein, recent applications and developments of machine learning, with a focus on deep learning, in virtual screening for active compound design are reviewed. This includes introducing different compound and protein encodings, deep learning techniques as well as frequently used bioactivity and benchmark data sets for model training and testing. Finally, the present state-of-the-art, including the current challenges and emerging problems, are examined and discussed.

## 1. Introduction

### 1.1. Virtual Screening

Drug discovery remains a key challenge in the field of bio-medicine. Traditionally, the discovery of drugs begins with the identification of targets for a disease of interest. It is followed by high-throughput screening (HTS) experiments to determine hits within the synthesized compound library, i.e., compounds showing promising bioactivity. Then, the hit compounds are optimized to lead compounds to increase potency and other desired properties, such as solubility, or vanishing toxic and off-target effects. After these pre-clinical studies, potential drug candidates have to pass a series of clinical trials to become approved drugs. On average, more than 2 billion US dollars and about 10–15 years are spent for developing a single drug [1]. While HTS experiments are very powerful, they remain time and cost-intensive, since they require several thousands of synthesized compounds, a large number of protein supplies, and mature methods for bioactivity testing in the laboratory [2].

To rationalize and speed up drug development, computational methods have been widely incorporated in the design process in the past three decades. One prominent method is virtual screening (VS), which is used to prioritize compounds from (ultra) large compound libraries which have a high potential to bind to a target of interest [3]. VS methods can efficiently scan millions of (commercially) available compounds, such as ZINC [4] or MolPORT [5], at low cost and prioritize those to be tested, synthesized in-house, or purchased from external suppliers. Besides, VS can be carried out in virtual compound libraries, which expands the chemical space, such as Enamine REAL [6] with over 17 billion make-on-demand molecules and a database containing close to two billion drug-like compounds. Although VS methods are not always able to find the most active compound, they can narrow the search space down to few hundreds of compounds with desired properties to be further investigated [7].

Nowadays, VS has become an integral part of drug discovery. It is usually implemented in the form of a hierarchical workflow, combining different methods (sequentially or in parallel) as filters to prioritize potentially active compounds [7,8]. VS methods are often divided into two major categories: 1. structure-based methods, which focus on the complementarity of the target binding pocket and the ligand; as well as 2. ligand-based methods, which rely on the similarity of novel compounds to known active molecules.

**Figure 1:**
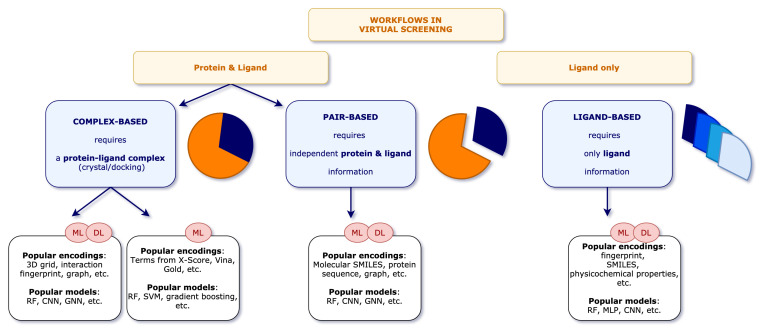
Workflows in virtual screening. The first split separates the schemes that contain (1) protein and ligand information and (2) ligand information only, which are typically used in models for QSAR predictions. For details on solely ligand-based methods, see for example MoleculeNet [25]. For (1), a second split makes the differences between complex-based and pair-based models. Complex-based models describe the protein and ligand in a complex, whereas pair-based models (also PCM in the broader sense) treat the protein and ligand as two independent entities. The latter typically use protein sequence and molecular SMILES information as input, while the complex-based models use, for example, a 3D grid of the protein-ligand binding site or interaction fingerprints.

Structure-based methods (1) require 3D structural information of both ligand and protein as a complex or at least of the protein with some knowledge about the binding site. The most commonly used technique is molecular docking, which predicts one or several binding pose(s) of a query ligand in the receptor structure and estimates their binding affinity [9]. While protein-ligand docking shows great ability in enriching likely active compounds over inactive ones, there are still complications in placing or scoring the individual poses, some of which can be unmasked by visual inspection [10,11,12,13]. During the molecular docking process, thousands of possible ligand poses are generated based on the target structure and ranked by a scoring function (SF) [14]. There are three classical types of scoring functions: physics-, empirical-, and knowledge-based [15,16]. Physics-based methods rely on molecular mechanics force fields. In short, non-bonded interaction terms such as Van der Waals interactions, electrostatics, and hydrogen bonds are summed. Similarly, empirical SFs sum weighted energy terms. Items describing for example rotatable bonds or solvent-accessible-surface area are also added and all terms are parameterized against experimental binding affinities. In contrast, knowledge-based methods rely on statistical analyses of observed atom pair potentials from protein-ligand complexes. More recently, new groups of scoring functions were introduced, namely machine/deep learning-based SFs. One group of models is based on classical SFs which try to learn the relationship between the interaction terms to predict binding affinity (see the review by Shen et al. [16]). Others models encode the complex via protein-ligand interaction fingerprints, grid- or graph-based methods [17]. Such models will be referred to as complex-based methods throughout this review and discussed in greater details, see Figure 1. Note that pharmacophore-based VS has also incorporated machine learning, and is suitable to screen very large databases, see for example Pharmit [18]. However, these methods are not the focus of this review and recent developments in the pharmacophore field are described by Schaller et al. [19].

Ligand-based methods (2), including QSAR (quantitative structure-activity relationship) modeling, molecular similarity search and ligand-based pharmacophores, are relatively mature technologies [20]. Unlike structure-based methods, ligand-based methods only require ligand information. Note that they are not the focus of this review and the reader is kindly referred to the respective literature, e.g., [20,21]. Nevertheless, the latter category can also be enriched by simple protein—mostly sequence-based—information and is often referred to as proteochemometric (PCM) modeling, which will be further addressed in this review. PCM combines both ligand and target information within a single model in order to predict an output variable of interest, such as the activity of a molecule in a particular biological assay [22,23]. Thus, PCM methods do not only rely on ligand similarities, but incorporate information from the target they bind to, and have been found to outperform truly ligand-based methods [24]. Note that in some PCM applications an additional cross-term is introduced that can describe the interaction between the two objects [22]. To distinguish the herein described methods, which handle the two objects individually, we refer to them as pair-based methods, see Figure 1.

### 1.2. Machine Learning and Deep Learning

Machine learning (ML) aims at learning a task from data by optimizing a performance measure. There exist three main approaches [26]: 1. Unsupervised learning in which the goal is to find patterns of the underlying structure and gain interpretability of the data. 2. Reinforcement learning in which an agent evolves in an environment and uses the data learned from experience. 3. Supervised learning in which an algorithm is trained on inputs to predict some labeled output. The latter technique will be the focus of this review.

Traditional supervised ML methods follow the idea that given some data, a predictive model is constructed by optimizing the difference between a given labeled output and the output predicted by the model. Some of these methods date back to the last century. For example, neural networks were first developed in the 60s by Rosenblatt [27]. Later, in the 80s, Breiman et al. [28] published the book *“Classification and regression trees“*. In the 90s, Cortes and Vapnik [29] introduced support vector machines (SVMs) and more recently, in the early 2000s, Breiman [30] proposed random forests (RFs).

Nevertheless, over the last few years, ML methods have gained a lot of popularity. This may be explained by three major aspects: 1. Data availability: thanks to automation and digitalization, as well as memory capacities, the amount of stored data has never been greater. 2. Computing power, such as graphics processing units (GPUs) and parallelization, has significantly allowed expensive model training. Cloud computing, for instance, Google Colaboratory [31], allows any user to train resource intensive machine learning models using powerful tensor processing units (TPUs). 3. The theoretical research on the learning algorithms has enabled the development of sophisticated models and training schemes.

Deep learning (DL) [32] is a subset of ML in which the input features are combined using hidden layers that constitute a network. Each hidden layer is made up of a linear and a non-linear part, the non-linear part called the activation function. The information then flows through the network. The resulting predictive model is highly flexible and is able to extract complex patterns thanks to the non-linearities. Since describing (and understanding) the interactions between molecular structures in a biological context is highly complex, it is not surprising that applying deep learning to such objects could yield excellent performance.

### 1.3. Data Availability and Big Data

As mentioned above, automation and storage have had a major impact on the amount of data existing nowadays. Recently, Google has published an image data set of over 9 million data points called “Open Images Dataset V4” [33] as well as “YouTube-8M”, a video data set of 8 million URLs. These large open-source data sets have enabled researchers to build highly efficient models in fields such as image classification. Benchmark data sets are also widely used in the machine learning community to train, test, and compare new models and architectures. One of the popular benchmark data sets in image classification is the MNIST database of handwritten digits [34] which has a training set of 60,000 examples and a test set of 10,000 examples. Kaggle [35] is a community that hosts competitions in very diverse fields, including drug activity prediction, where the data are made public. These competitions allow to prospectively evaluate all kinds of different schemes and rank them using hold out data sets.

In the biomedical field, the size of the data sets is starting to reach similar scales. The amount of publicly available bioactivity data keeps increasing every year. To date, the well-known ChEMBL database [36] has 17,276,334 registered activity entries [37] and has become a valuable resource in many types of life science research. Furthermore, a considerable amount of structural data has also been published over the last decades. The freely available Protein Data Bank (PDB) [38,39] logged 14,047 new entries in 2020. In March 2021, the total number of available entries has surpassed 175,000 [40] and will most probably keep increasing. The structural data come from experimental methods, such as X-ray crystallography, nuclear magnetic resonance spectroscopy and electron microscopy, technologies that have improved in precision and throughput over the last years [41,42]. Publicly available screening libraries also have big data potential. For example, the ZINC database [4] contains over 230 million of commercially available compounds. More details on specific data sets will be given in Section 2.

### 1.4. Deep Learning in Virtual Screening

Given the increasing amount of available structural and bioactivity data as well as the recent progress in machine—especially deep—learning, it is no wonder that virtual screening strategies could benefit from this synergy.

While ML methods have been applied in the field for over two decades already [43,44,45], DL has begun to rise in the drug discovery area, especially in VS [46]. Given the new developments, various reviews about ML and DL in VS have recently been published [16,47,48,49,50,51,52]. For example, Shen et al. [16] and Li et al. [50] review differences between more traditional ML—and DL—based scoring functions (SFs). Rifaioglu et al. [52] present an overview of recent applications of DL and ML on in silico drug discovery. In contrast, this review focuses on the one hand on advances regarding DL-based VS in recent years, and on the other hand covers two main groups of models, both including information from the protein and the ligand: 1. Complex-based models, which are trained on information/encodings from complexes or docking poses of protein and ligand for predicting the binding affinity of a given molecule; and 2. pair-based models or PCM, which are primary ligand-based but include simple information from the protein they bind to.

## 2. Methods & Data

In this section, the main encodings of ligand, protein and complex, the different deep learning models as well as the most used (benchmark) data sets are introduced.

### 2.1. Encodings in Virtual Screening

The interactions between protein and ligands are complex, and encoding the most informative bits in a computer-readable format is one of the main challenges in both cheminformatics and bioinformatics. In the following sections, the encodings for ligands in virtual screening are described, followed by protein and complex encodings. The details are reported for those used in the studies discussed in Section 3. A more exhaustive list of ligand encodings is carefully outlined in the review by Lo et al. [53]. For protein descriptors, the work by Xu et al. [54] describes common sequence- as well as structure-based descriptors, embedding representations and possible mutations.

#### 2.1.1. Ligand Encodings

**Figure 2:**
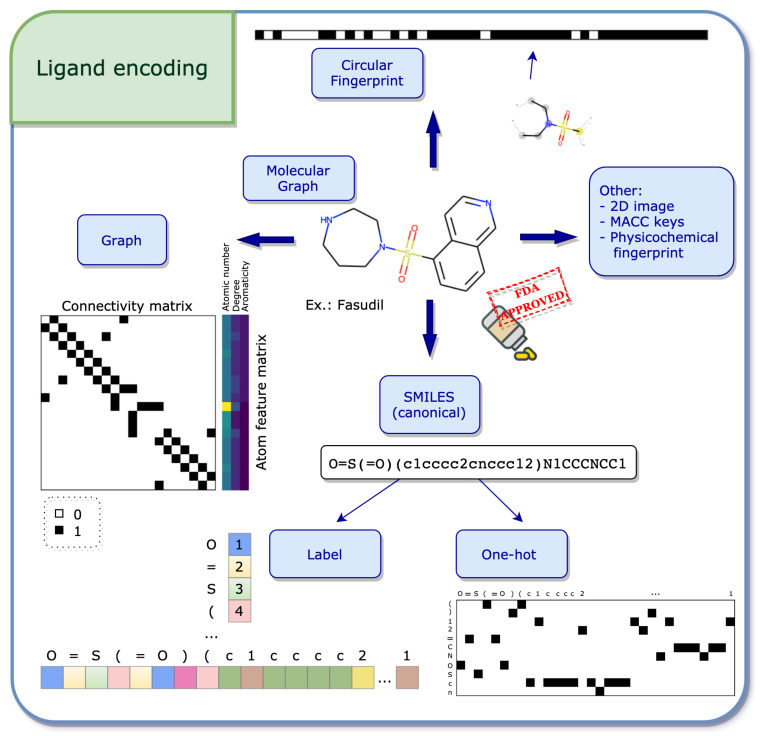
Ligand encoding. Having a computer-readable format is one of the starting points for machine—and deep—learning. The example molecule is the FDA–approved drug Fasudil [55] taken from the PKIDB database [56]. Recent studies focused on virtual screening (detailed in Section 3) commonly use SMILES, circular fingerprints or graphs to encode the ligand.

The starting point of several ligand encodings is the molecular graph, where nodes and edges represent the molecular atoms and bonds, respectively (see Figure 2).

Graph

The molecular graph can be encoded using two matrices: the first one, called the feature matrix *X*, gives a per atom description, where the type of information stored in each node is decided a priori. Common per atom examples are atomic type and degree [57]. The dimension of *X* is N×D, where *N* is the number of nodes, i.e., atoms, in the graph and *D* the number of pre-defined features. The second matrix, called connectivity matrix, describes the structure of the molecule. Its purpose is to illustrate how the nodes are connected in the graph, i.e., via bonds. Two frequent formats store this information: 1. the adjacency matrix *A* of dimension N×N, where $A_{ij}=1$ if node *i* is connected to node *j* and 0 otherwise.2. The coordinate (COO) format of dimension 2×E, where *E* represents the number of edges in the graph. Thus, the first and second rows represent the index of the source and target nodes, respectively. Using both the feature matrix and the connectivity matrix, the molecular graph encoding can further be used to apply machine learning algorithms.

SMILES

An efficient way of storing information from the molecular graph using string characters is the simplified molecular input line entry system (SMILES) developed by Weininger [58]. The main idea behind SMILES is the linearization of the molecular graph by enumerating the nodes and edges following a certain path. Due to the randomness in the choice of the starting atom and the path followed along the 2D graph, there exist several valid SMILES for one molecule [59]. However, it may be desirable to have one unique SMILES for a given compound, called the canonical SMILES, and most software have their own canonization algorithm. In order to apply mathematical operations in the context of machine learning, SMILES still need to be transformed into numerical values, where both label and one-hot encoding are often used [60,61]. Please find more information on these encodings below.

Label and One-Hot Encoding

In this section, the concepts of label and one-hot encoding are explained in the context of SMILES, but the idea can be translated to broader applications, such as natural language processing (NLP) [32]. Mathematical operations such as matrix computations cannot be applied directly to string characters and therefore these strings have to be transformed into numerical entities. The first step towards this transformation is the specification of a dictionary of considered characters, which can be done in two ways: either by inserting all the characters existing in the data set, or by exhaustively enumerating a list of all necessary characters. Once the dictionary is defined, label or one-hot encoding can be used.

**Figure A1:**
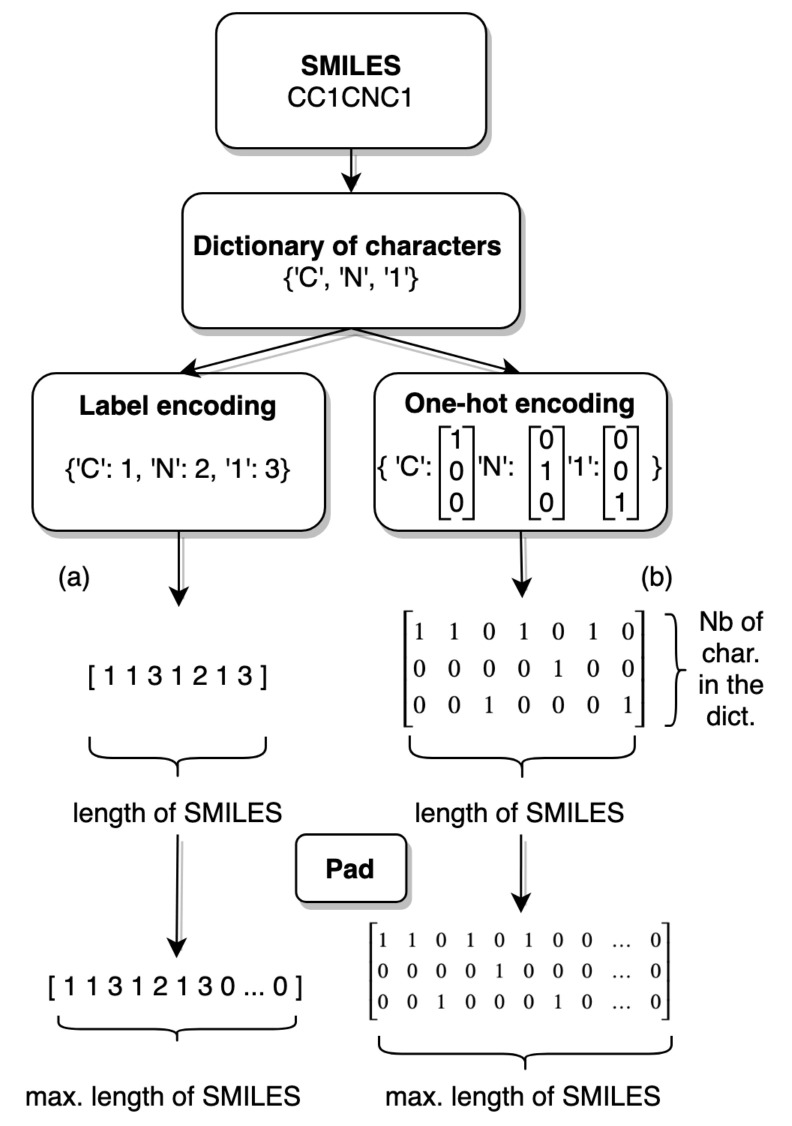
Encoding and padding. Starting from a SMILES string, the characters can be stored in a dictionary (or the dictionary can be constructed prior using a set of known characters). Label encoding consists of enumerating the characters in the dictionary. One-hot encoding consists of assigning a binary vector to each character. (**a**) Constructing the label encoding for a given SMILES by assigning the integer associated to the character as they appear in the SMILES. (**b**) Constructing the one-hot encoding by concatenating the binary vectors of the characters as they appear in the SMILES. (Pad) Inputs of same dimension are often required when using machine learning. A common solution is to use padding, which consists of adding zeros to either the label vector or to the one-hot matrix up to the maximum length of the SMILES in the data set.

In label encoding, the characters in the dictionary are enumerated. This enumeration using integer numbers is also sometimes referred to as integer encoding. Using the integer labels, a SMILES can be transformed into an integer vector by associating each character with its integer label (see Figure A1a). The advantage of such a representation is its compact form, leading to a simple integer vector. A disadvantage however is the natural hierarchy in the numbering, giving higher values to some characters in the dictionary.

The one-hot encoding resolves this issue by assigning a binary vector to each character in the dictionary. A SMILES can then be constructed by concatenating the binary vectors as they appear in the SMILES (see Figure A1b). The main disadvantage of using the one-hot transformation is that the resulting matrix may be large and sparse. In both label and one-hot encoding, having all elements in a data set with the same dimension is often required in a machine learning setting. A way to account for the different dimensions is to use padding, which adds zeros to the vector (in the label encoding case) or to the matrix (in the one-hot encoding case) up to a maximum length, usually determined by the longest element in the data set.

Circular Fingerprint

Circular fingerprints are, once folded, fixed-length binary vectors that determine the presence (encoded by 1) of a substructure or the absence of it (encoded by 0). The recursive algorithm behind extended-connectivity fingerprints (ECFP) [62] starts with an atom initializer for each node and updates the atom identifiers using a hash function by accumulating information from neighboring nodes. The substructures which are identified using the local atom environments correspond to the bits in the fingerprints. A free version of the algorithm is available in the open-source cheminformatics software RDKit [63] (under the name of Morgan fingerprints), which will not produce the same results as the original implementation in Pipeline Pilot [64] due to the difference in the hash functions, but will yield similar results.

Other Encodings

Ligands are evidently not restricted to these encodings [53]. For example, different types of fingerprints may be used, such as a physicochemical-based vector, describing the global properties of the molecule, as in the study by Kundu et al. [65]. Also, the 166-bit long MACCS keys [66] are a common way to encode molecular compounds as a fingerprint. Recently, learned fingerprints have also shown to be effective in QSAR predictions [61,67]. Another way of employing the molecular structure as input to machine learning is the 2D image itself. Rifaioglu et al. [68] use the 200-by-200 pixel 2D image generated directly from the SMILES using the canonical orientation/depiction as implemented in RDKit [63].

#### 2.1.2. Protein Encodings

Proteins are macromolecules that are involved in many biochemical reactions. They are composed of distinct amino acid sequences, which result in folding in specific 3D protein structures [69].

Protein Identifier

A simple way to discriminate models from ligand information only is to include the identifier (ID) of the protein. Such a descriptor adds no information whatsoever about the physicochemical properties of the protein, the amino acid composition, nor the 3D conformation. It is merely a way for a machine learning model to be able to differentiate several proteins. For example, the one-hot encoding of the protein ID can be used, as in the study by Sorgenfrei et al. [70].

Protein Sequence

The (full) sequence of a protein, often referred to as the primary structure, is the enumeration of the amino acids as they appear from the beginning (N-terminus) to end (C-terminus) of the protein, in which each of the 20 standard amino acids can be encoded as a single letter. The length of a protein can vary greatly, some of them containing thousands of amino acid residues. Although the sequence is a compact way of storing information about the primary structure, it does not give any information about the 3D structure of the protein. In opposition to the full sequence, it is possible to only consider the sequence from the binding site, reducing greatly the number of residues.

Z-Scales

The z-scale descriptors published in the late 80s by Hellberg et al. [71] are constructed by considering, for each of the 20 amino acids, 29 physicochemical properties such as the molecular weight, the logP and the logD (see [70], Table 1). A principal component analysis (PCA) on the 20×29 matrix is performed and the three principal components $z_{1}$, $z_{2}$ and $z_{3}$ for each of the amino acids are retained. The authors suggest interpreting $z_{1}$, $z_{2}$ and $z_{3}$ as hydrophilicity, bulk and electronic properties, respectively.

Domains and Motifs

A domain is a structural unit within a protein that is conserved and the overall role of a protein is often governed by its domain function. PROSITE [72] and Pfam [73] are two popular databases that store a large variety of protein domains. Therefore, a possible way of encoding proteins is through a binary vector which indicates the presence or absence of a particular domain.

Structural Property Sequence

In the study by Karimi et al. [74], the proteins are encoded using structural property sequences, which describe the structural elements of the protein but do not require the 3D structures. The secondary structure is predicted from the sequence using SSpro, developed by Magnan and Baldi [75]. Neighboring residues are thereby grouped together to form secondary structure elements. Then, four letters are assigned to each of these elements. The first letter represents the secondary structure: alpha helix (A), beta sheet (B) or coil (C). The second letter determines the solvent exposure: N as “not exposed” or E as “exposed”. The third letter describes thy physicochemical properties, i.e., non-polar (G), polar (T), acidic (D) or basic (K). The last letter represents the length: small (S), medium (M) or large (L).

#### 2.1.3. Complex Encodings

**Figure 3:**
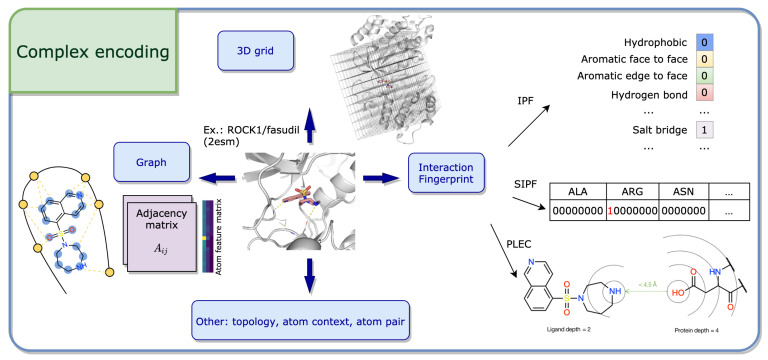
Complex encoding. Visual representation of encodings for protein-ligand complexes used in structure-based virtual screening, exemplified with the drug Fasudil co-crystallized with the ROCK1 kinase (PDB ID: 2esm). 3D grids, graphs and interaction fingerprints are among popular encodings for complexes, as discussed in Section 3.

Describing the protein-ligand complex involves descriptions that capture the interactions between the two binding partners. Herein, we group them into interaction fingerprints, 3D grids, graphs and other, see Figure 3.

Interaction Fingerprint

Interaction fingerprints (IFPs) describe—as the name implies—the interactions between a protein and a ligand based on a defined set of rules [21,76]. Typically, the IFP is represented as a bit string, which encodes the presence (1) or absence (0) of interactions between the ligand and the surrounding protein residues. In most implementations, each binding site residue is described by the same number of features, which usually include interaction types such as hydrophobic, hydrogen bond donor and acceptor.

*IFPs encoding interaction types:* The structural interaction fingerprint (SIFt) [77] describes the interactions between the ligand and *n* binding site residues as an n×7 long bit string. Here, the seven interaction types include whether the residue (i), and more precisely their main (ii) or side (iii) chain atoms, are in contact with the ligand; whether a polar (iv) or apolar (v) interaction is involved; and whether the residue provides hydrogen bond acceptors (vi) or donors (vii). Similarly, the protein-ligand interaction fingerprint (PyPLIF) [78] and the IChem’s IFP [79] encode each residue also by seven though slightly different types, while PADIF [80] uses the Gold [81] scoring function contributions as interactions types.

The aforementioned IFPs vary in size and are sensitive to the order of the residues, which limits their application for ML purposes. Thus, SILRID [82], a binding site independent and fixed-length IFP was introduced. SILRID generates a 168 long integer vector per binding site, obtained by summing the bits corresponding to a specific amino acid or cofactor (20+1), while each amino acid is described by eight interaction types.

*IFPs including distance bits:* To describe the interactions more explicitly, distances with respect to interaction pairs or triples were introduced. APIF [83], an atom-pair based IFP, encodes three interaction types for the protein and ligand atoms: hydrophobic contact, hydrogen bond donor and acceptor. Combinations of these three types lead to six pairings, including for example a protein acceptor-hydrophobic pair complemented with a ligand donor-hydrophobic atom-pair. Moreover, for each pairwise interaction in the active site, the respective receptor and ligand atom distances are measured and binned into seven ranges. In this way, the total APIF is composed of 6 types ×7 protein distances ×7 ligand distances =294 bits. Pharm-IF [84], while using slightly different interaction type definitions, calculates distances between the pharmacophore features of their ligand atoms. Finally, triplets between interaction pseudoatoms are introduced in TIFP [85]. The fingerprint registers the count of unique interaction pseudoatom triplets encoded by seven properties (such as hydrophobic, aromatic or hydrogen-bond) and the related distances between them, discretized into six intervals. Redundant and geometrically invalid triplets are removed, and the fingerprint is pruned to 210 integers representing the most frequently occurring triplets in the appointed data set.

*IFPs including circular fingerprint idea:* To become more independent of pre-defined interaction types, circular fingerprint inspired IFPs were introduced by encoding all possible interaction types (e.g., π−π,CH−π) implicitly via the atom environment. The structural protein-ligand interaction fingerprint (SPLIF) [86] is constructed using the extended connectivity fingerprint (ECFP, see Section 2.1.1 for more information). For each contacting protein-ligand atom pair (i.e., distance less than 4.5 Å), the respective protein and ligand atoms are each expanded to circular fragments using ECFP2 and hashed together into the fingerprint. Similarly, ECFP is integrated in the protein-ligand extended connectivity (PLEC) fingerprint [87], where *n* different bond diameters (called “depth”) for atoms from protein and ligand are used.

3D Grid

Another type of encoding are 3D grids, in which the protein is embedded into a three-dimensional Cartesian grid centered on the binding site. Similar to pixel representation in images, each grid point holds one (or several) values that describe the physicochemical properties of the complex at this specific position in 3D space. Such grids can, for example, be unfolded to a 1D floating point array [88] or transformed into a 4D tensor [89] as input for a DL model. Depending on the implementation, the cubic grids vary in size between 16 Å and 32 Å, as well as grid spacing (resolution) usually being either 0.5 Å or 1 Å [88,89,90,91]. Per grid point attributes can be: 1. simple annotations of atom types or IFPs, as in AtomNet [88] and DeepAtom [92], 2. physicochemical or pharmacophoric features, as in Pafnucy [89] and BindScope [93], or 3. energies based using one or several probe atoms as in AutoGrid/smina [90,94].

Graph

Although the description of a small molecule as a graph seems natural, the idea can be adapted to a molecular complex. As in the ligand case (see Section 2.1.1), two main components have to be considered in the graph description of such protein-ligand structures: the nodes, with an associated feature vector, and the relationship between them, usually encoded in matrix form. When considering a complex, the atoms from both the protein and the ligand can simply be viewed as the nodes of the graph and the atomic properties can vary depending on the task at hand. Some might consider, among other characteristics, the one-hot encoded atom type/degree and a binary value to describe aromaticity, as in [95]. As simple as the node description is for complexes, the intricacy arises when describing the interactions between the atoms, which should account for covalent and non-covalent bonds. The focus here will be on two different ways of describing such structures. The first one, developed by Lim et al. [95], considers two adjacency matrices $A^{1}$ and $A^{2}$. $A^{1}$ is constructed in such a way that it only takes into account covalent bonds, more precisely $A_{ij}^{1}=1\;\mathrm{if}\;i,j$ are covalently connected, and 0 otherwise. $A^{2}$, on the other hand, not only captures bonded intramolecular and non-bonded intermolecular interactions, but also their strength through distances. Mathematically, this can be translated as follows: if atom *i* belongs to the ligand, atom *j* to the protein, and they live in a neighborhood of 5 Å, then

$$A_{ij}^{2}=e^{-}\frac{{\left(d_{ij}-\mu \right)}^{2}}{\sigma },$$

where $d_{ij}$ is the distance between atoms *i* and *j*, and μ and σ are learned parameters. The smaller the distance between the atoms to μ is, the stronger the bond is. If atoms *i* and *j* both belong to either the ligand or the protein, then $A_{ij}^{2}=A_{ij}^{1}$.

The other graph form of protein-ligand developed by Feinberg et al. [96] consists of an enlarged adjacency matrix $A\in R^{N\times N\times N_{et}}$, where *N* is the number of atoms and $N_{et}$ the number of edge types. $A_{ijk}=1$ if atom *j* is in the neighborhood of atom *i* and if *k* is the bond type between them. If not, that same entry is 0. This scheme numerically encodes the spatial graph as well as the bonds through edge type.

Other Encodings

Moreover, there are also other encoding methods to describe a complex, which will only be shortly introduced here. Topology-based methods, as reported by Cang and Wei [97], describe biomolecular data in a simplified manner. The topology thereby deals with the connectivity of individual parts and characterizes independent entities, rings and higher dimensional faces. In this way, element-specific topological fingerprints can retain the 3D biological information and the complex can be represented by an image-like topological representation (resembling barcodes).

Also, simply the protein-ligand atom pairs together with their distances can be used as input. In the work by Zhu et al. [98], all atom pair energy contributions are summed, where the contributions themselves are learned through a neural network considering the properties of the two atoms and their distances. Similarly, Pereira et al. [99] introduced the atom context method to represent the environment of the interacting atoms, i.e., atom and amino acid embeddings.

### 2.2. Deep Learning Models in Virtual Screening

As mentioned in the introduction, machine learning can be split into supervised, unsupervised and reinforcement learning. In this section, we focus on supervised learning which is a framework that is used when the data is constituted of some input and an associated label and the aim is to predict the outcome corresponding to a given input. Subsequently, typical evaluation strategies of machine learning models will shortly be introduced.

#### 2.2.1. Supervised Deep Learning Models

In the supervised framework, two subclasses are usually considered: the first one, called classification, deals with discrete outputs. In the binary case, this reduces to outputs that can take either 0 or 1 values. In the context of virtual screening, a simple example would be the activity determination of a compound against a protein: active (1) or inactive (0). The second subclass is regression, where the target value takes a real value instead. An analogous example in VS would be to predict the half maximal inhibitory concentration $IC_{50}$ of a compound. The $IC_{50}$ describes the amount of a substance that is needed to inhibit a target protein/assay by 50%.

**Figure 4:**
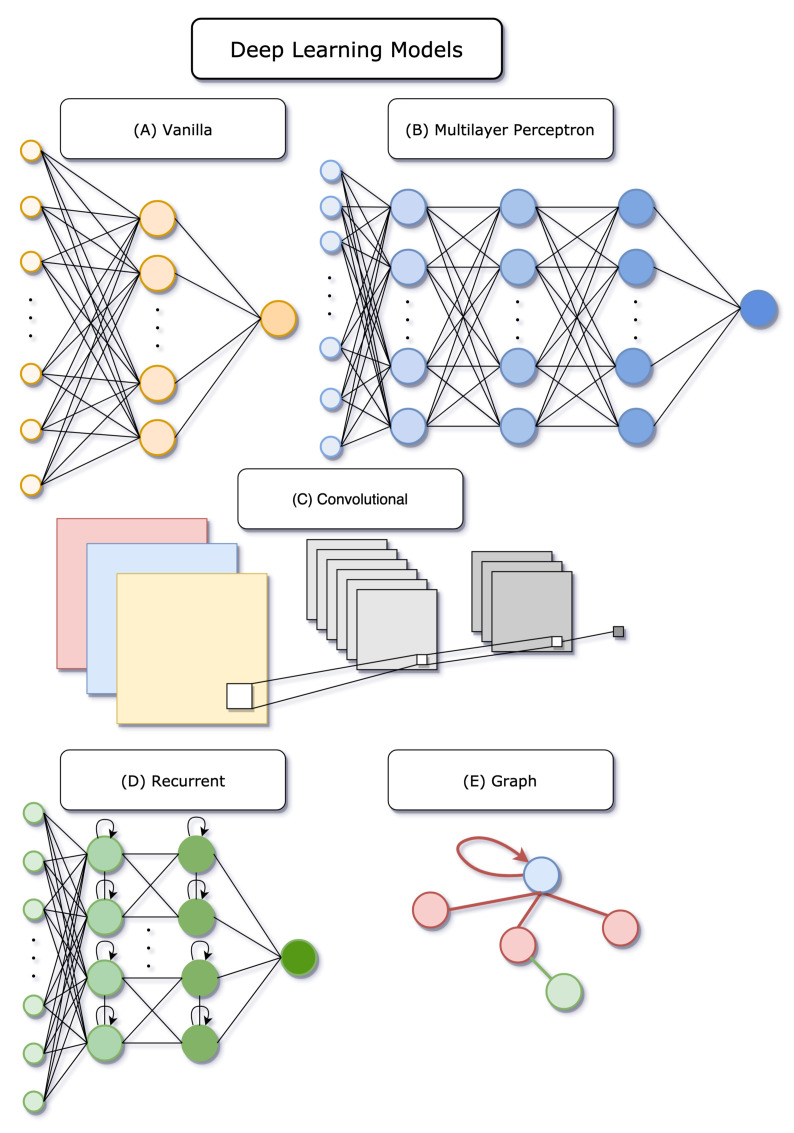
Deep learning models. Schematic illustration of the neural networks described in Section 2.2. (**A**) Vanilla neural network. (**B**) Multilayer perceptron with three hidden layers (MLP). (**C**) Convolutional neural network (CNN). (**D**) Recurrent neural network (RNN). (**E**) Graph neural network (GNN). CNNs and GNNs particularly have become very popular in recent virtual screening studies (see tables in Section 3).

Common machine learning algorithms include tree-based methods such as random forests (RFs), tree boosting, and support vector machines (SVMs). However, over the last decades, deep learning has gained a lot of momentum and the rest of this section will be dedicated to the idea behind the deep learning models described in Section 3 (see Figure 4). For more rigorous definitions and mathematical notations, the reader is kindly referred to the book by Goodfellow et al. [32].

##### 2.2.1.1. Neural Networks

Neural networks (NNs) [32], also sometimes called artificial neural networks (ANNs), are models that take as input a set of features on which mathematical computations are performed that depend on a set of parameters. The sequential computations between the input and the output are called hidden layers and the final one, the last layer, should account for the targeted prediction: classification or regression. The information flows through the network and is monitored by non-linearities called activation functions that determine if or how much of the information can be passed on to the next layer. The parameters in the network are optimized using back-propagation ([32], Chapter 6).

A simple example of a neural network connects the input to the output with one single hidden layer and is sometimes called a “vanilla network” or a “single layer perceptron” [100], in opposition to a multilayer perceptron (MLP) that has more than one hidden layer. In a single layer perceptron, the hidden layer is composed of a set of nodes where each input element is connected to every hidden node and every node in the hidden layer is connected to the output. When all nodes from one layer are connected to the next, the layer is called fully-connected, or dense. If the network contains only such layers, then it is usually referred to as a fully-connected neural network, a dense neural network, or a multilayer perceptron. Note that throughout this review, the term MLP is used, while in the original publications other terms might be preferred. Such models can be easily applied to a set of vectors with a corresponding target value, as exemplified for chemical compound fingerprints and associated cytotoxicity values by Webel et al. [101].

##### 2.2.1.2. Convolutional Neural Networks

Convolutional neural networks (CNNs) ([32], Chapter 9) are a special kind of network where computations in the hidden layer make use of convolutions. They are most commonly applied in image classification, where their forte is extracting features in a picture, such as edge detection [102,103,104]. 1D, 2D, and 3D convolutions can be used depending on the input data. In the one-dimensional case, the data might be akin to time series. 2D convolutions are used on planar grid-like structures, such as images. 3D convolution can be applied to three-dimensional tensors, such as 3D images. Although convolutional neural networks exhibit excellent performance, they have the disadvantage of having a fast-growing number of parameters as the network becomes deeper, especially in the 3D case, making the training slow. In the area of binding affinity, successful predictions have been made using as input the 3D representation of the protein-ligand binding site [91].

##### 2.2.1.3. Recurrent Neural Networks

Recurrent neural networks (RNNs) ([32], Chapter 10) are dissimilar from MLPs and CNNs in their ability to reuse internal information, that can be thought of as loops in the network (see Figure 4D). RNNs are well suited for sequential data, such as sentences. The input at a certain time makes use, employing a series of computations, of the input at the previous time, leading to its name “recurrent”. In molecular prediction, SMILES encoding of molecules can be interpreted as sequential data and RNNs were successfully applied in the QSAR context [59].

##### 2.2.1.4. Graph Neural Networks

Graph neural networks (GNNs) [105] are models that require graph-structured data. Loosely put, the input should represent a set of vertices (or nodes) and some structure that determines the relationship between them. The graph neural network will act on the nodes while taking into account the neighbors of each node, i.e., the set of vertices that are connected to that particular node. Each node is updated to a latent feature vector that contains information about its neighborhood, hence resembling a convolution operation and leading to the denomination of graph convolution neural network (GCNN). This latent node representation, often called an embedding, can be of any chosen length. These embeddings can further be used for node prediction, if some properties of vertices are of interest, or they can be aggregated to obtain predictions at the graph level, i.e., some information on the graph as a whole.

A subtlety that can be added to GNNs is a gated recurrent unit (GRU), which is a way to moderate the flow of information coming from other nodes and from previous computation steps [106]. These particular units are often applied at an intermediary stage, once the embeddings from previous steps are set. GRUs consists of two gates: the update gate is responsible for updating the weights and biases and the reset gate for controlling the amount of information that can be forgotten. Graph networks using GRUs are called gated graph neural networks (GGNNs) [106].

Graph attention neural networks (GANNs) [107] are graph neural networks with an added attention mechanism. In a graph setting, this can be viewed as ranking the nodes in a neighborhood of a given vertex and giving more or less importance to each of them. Certain atoms, and therefore interactions, may have more significance for a given task. This can be represented by including distances between atoms in the adjacency matrix, as in [95]. A feature node is then obtained using a linear combination of its neighbors taking the attention coefficient into account.

More details on graph neural networks can be found in the review by Zhou et al. [108]. In the context of molecular prediction, dozens of examples use GNNs, as summarized in the review by Wieder et al. [109].

#### 2.2.2. Model Evaluation Strategies and Metrics

To assess the performance of any machine learning method, the data is commonly split into training and test sets. The model is trained on the training set and evaluated by comparing the predicted labels to the given labels on the hold out (test) set. Here, the metrics used in Section 3 are simply listed, for a detailed description please refer to Appendix B.

For regression tasks, the metrics reported are the mean squared error (MSE) and the root mean squared error (RMSE). For classification tasks, the area under the ROC—receiver operating characteristic—curve (AUC), the accuracy or the enrichment factor (EF) are used. For both regression and classification, the Pearson correlation coefficient *R*, the Spearman’s correlation coefficient ρ or the coefficient of determination $R^{2}$ may be reported.

Cross-validation (CV) is very often used to estimate the prediction error and usually performed using five or ten folds, and the results are reported as mean performance (±standard deviation). Additionally, CV can be used for hyper-parameter tuning. Please refer to the work by Hastie et al. [100] for a full description of this method.

### 2.3. Data Sets and Benchmarks in Virtual Screening

The quality and quantity of data sets in the biomedical field have increased largely over the last years, boosting the usage of ML and DL models in drug discovery. The main source of freely available 3D structural information of proteins as well as protein-ligand complexes is the well-known Protein Data Bank (PDB) [38], holding, as of March 2021, 175,282 biological macromolecular structures [40], a number which includes proteins that have been solved numerous times. Furthermore, labeled bioactivity data, i.e., the measured activity of a specific compound against a target of interest, are necessary for training, validating, and testing DL models. The two most well-known examples of bioactivity databases are PubChem [110] and ChEMBL [36]. Note that while for pair-based methods, the information in the latter databases is sufficient, for complex-based methods the bioactivity and structural information has to be linked. Below, the most widely used labeled bioactivity data sets and their composition will be introduced (see Table 1).

#### 2.3.1. Structure-Based Data Sets

##### 2.3.1.1. PDBbind

The PDBbind [111] database collects experimentally measured binding affinity data from scientific literature for a large number of biomolecular complexes deposited in the PDB database. In the current release, PDBbind v.2019 provides binding data of a total of 21,382 biomolecular complexes as the general set, including 17,679 protein-ligand complexes. Furthermore, a refined and a core set with higher quality data are extracted from the general set. In the refined set, the 4852 protein-ligand complexes meet certain quality criteria (e.g., resolution, R-factor, protein-ligand covalent bonds, ternary complexes or steric clashes, and type of affinity value). The core set, resulting after further filtering, provides 285 high-quality protein-ligand complexes for validating docking or scoring methods.

##### 2.3.1.2. BindingDB

BindingDB [112] is a publicly accessible database, which collects experimental protein-ligand binding data from scientific literature, patents, and other. The data extracted by BindingDB includes not only the affinity, but also the respective experimental conditions (i.e., assay description). BindingDB contains 2,229,892 data points, i.e., measured binding affinity for 8499 protein targets and 967,208 compounds, including 2823 protein-ligand crystal structures with mapped affinity measurements (requiring 100% sequence identity), as of 1 March 2021 [113].

##### 2.3.1.3. BindingMOAD

BindingMOAD (Mother of All Databases) [114,115] is another database focused on providing combined high-quality structural and affinity data, similar to PDBbind. BindingMOAD (release 2019) contains 38,702 well-resolved protein-ligand crystal structures, with ligand annotation and protein classifications, of which 15,964 are linked to experimental binding affinity data with biologically-relevant ligands.

#### 2.3.2. Bioactivity Data Sets

##### 2.3.2.1. PubChem BioAssay

PubChem [110] is the world’s largest freely available database of chemical information, e.g., chemical structures, physicochemical properties, biological activities, patents, health, safety and toxicity data, collected from more than 770 data sources (as of March 2021 [116]). PubChem BioAssay contains bioactivity data, more precisely biological assay descriptions and test results, for compounds and RNAi reagents assembled from high-throughput screens and medical chemistry studies. As of March 2021, PubChem deposited more than 109 million unique chemical structures as well as over 280 million bioactivity data points collected from more than 1.2 million biological assays experiments.

##### 2.3.2.2. ChEMBL

ChEMBL [36,117] is a widely used open-access bioactivity database with information about compounds and their bioassay results extracted from full-text articles, approved drugs and clinical development reports. The last release, ChEMBL v.28, contains 14,347 targets and over 17 million activities, which are collected from more than 80,000 publications and patents [37], alongside deposited data and data exchanged with other databases such as BindingDB and PubChem BioAssay.

##### 2.3.2.3. Target-Family Specific Data Sets (Such as Kinases)

Since some protein families sparked a special interest in pharmaceutical sciences due to their central therapeutic role, target-family specific data sets have been composed. Kinases, for example, play a major role in many diseases and have been extensively studied, also computationally [118], for drug design. Data sets comprise profiling studies, such as the one reported by Davis et al. [119], which provides information about a full matrix of 72 inhibitors tested against a set of 442 kinases in competition binding assays (measured dissociation constant $K_{d}$). To be able to combine data from different sources, reported as different bioactivity measurements (e.g., $IC_{50}$, $K_{d}$ and inhibition constant $K_{i}$), Tang et al. [120] derived a kinase inhibitor bioactivity (KIBA) score, an adjusted Cheng-Prusoff model, which allows to integrate data from the above mentioned measurement types and to assemble a freely available drug–target bioactivity matrix of 52,498 chemical compounds and 467 kinase targets, including 246,088 KIBA scores.

#### 2.3.3. Benchmarking Data Sets

The above introduces data sets commonly used for training ML models in the context of VS. Nevertheless, defined benchmarking data sets are needed for a standardized comparison among different methods and studies [121]. Here, frequently used benchmarking data sets for structure- and ligand-based VS are introduced (see Table 2).

##### 2.3.3.1. CASF

The comparative assessment of scoring functions (CASF) benchmark [122] is developed to monitor the performance of structure-based scoring functions. In the latest version, CASF-2016, the PDBbind v.2016 core set was incorporated with 285 high-quality protein-ligand complexes assigned to 57 clusters. Scoring functions can be evaluated by four metrics: 1. The scoring power, indicating the binding affinity prediction capacity using the Pearson correlation coefficient *R* [123]. 2. The ranking power, showing affinity-ranking capacity using the Spearman correlation coefficient ρ [124,125]. 3. The docking power, using the root mean square deviation (RMSD) [126] to analyze how well the method has placed the ligand (pose prediction). 4. The screening power measures the enrichment factor (EF) [127], showing the ability of the function to prioritize active over inactive compounds.

Note that the CASF team has evaluated scoring functions from well-known docking programs, such as AutoDock vina [128], Gold [81], and Glide [129], and published the results on their website [122].

##### 2.3.3.2. DUD(-E)

The directory of useful decoys (DUD) [130] is a virtual screening benchmarking set providing 2950 ligands for 40 different targets, and 36 decoy molecules per ligand drawn from ZINC [4]. Decoys, i.e., negative samples, are chosen to have similar physicochemical properties, but dissimilar 2D topology to the respective active molecules. DUD-E [131] is an enhanced and rebuilt version of DUD, with 22,886 active compounds and affinity values against 102 diverse targets. On average, 50 decoys for each active compound are selected. DUD-E is usually used in classification tasks to benchmark molecular docking programs with regard to their ability to rank active compounds over inactive ones (decoys).

##### 2.3.3.3. MUV

The maximum unbiased validation (MUV) data set [132] is based on the PubChem BioAssay database mostly for ligand-based studies, using refined nearest neighbor analysis to select actives and inactives, to avoid analogue bias and artificial enrichment. It contains 17 different target data sets, each containing 30 actives and 15,000 inactives. Note that in contrast to DUD(-E) decoys, the inactives have experimental validated activities.

##### 2.3.3.4. Benchmarking Set Collections

Note that several collections of data sets for the purpose of benchmarking molecular ML models, with focus on model architectures and/or encodings, have recently been made freely available. These include, but are not limited to: 1. MoleculeNet [25] to benchmark molecular machine learning, currently providing a total of 700,000 compounds tested on diverse properties from not only quantum mechanics, but physical chemistry, biophysics (including MUV and PDBbind) and physiology; 2. Therapeutics Data Commons (TDC) data sets [133], including 22 machine learning tasks and 66 associated data sets covering various therapeutic domains; or 3. the work by Riniker and Landrum [134], covering compounds for 118 targets from MUV, DUD and ChEMBL with focus on benchmarking fingerprints in ligand-based virtual screening.

## 3. Recent Developments

In this section, recent developments in virtual screening (VS) are described and specifically how deep learning (DL) helps to improve drug-target binding, i.e., activity/potency prediction. Our review focuses on methods using protein and ligand information (see Figure 1), either in form of a protein-ligand complex (complex-based) or considering protein and ligand as two independent entities (pair-based/PCM). It is imperative to state that the aim of this section is not to directly compare different studies or models, but to describe them and put them into context. The list of abbreviations can be found at the end of this review.

### 3.1. Complex-Based Models

In this section, recent methods that require complex structure information, usually explicitly or implicitly described by the interactions between the protein and the ligand, will be discussed (see Table 3). The various methods are grouped by the type of encodings used for the complex structure: IFPs, 3D grids, graphs and other (see Section 2).

Interaction Fingerprint-Based Studies

Interaction fingerprints, which are often used for binding site comparison [21,142], have also been successfully applied to VS. Due to the difference in length of some IFP implementations, binding site independent IFPs are more commonly used for machine learning applications.

In 2009, Sato et al. [84] combined machine learning (among others SVM, RF, and MLP) and the pharmacophore-based interaction fingerprint (Pharm-IF) are incorporated for screening five selected protein data sets in silico. For training a model per protein, the respective co-crystallized ligands served as active samples (between 9 and 197) and the Glide docking poses of 2000 randomly selected decoys from PubChem as negative samples. For the test set, 100 active samples (after clustering) were drawn from the StARlite database (data now contained in ChEMBL) together with 2000 negatives samples (as above) and all compounds were docked using Glide. The combination of SVM and Pharm-IF performed best with a high mean $EF_{10\%}$ of 5.7 (over the five protein sets) compared to Glide scores (4.2) and a residue-based IF (PLIF) model (4.3), as well as a high mean AUC value of 0.82 compared to Glide (AUC 0.72) and PLIF model (AUC 0.78). Interestingly, in this study, the Pharm-IF SVM model outperformed the respective MLP model (average $EF_{10\%}$ 4.42, AUC 0.74). In 2016, Wang et al. [135] used ensemble learning to improve the SVM performance (using Adaboost-SVM) with the Pharm-IF encoding for two proteins from the same data set and gained even higher $EF_{10\%}$ values.

In 2019, Li et al. [136] introduced an application of a target-specific scoring model to identify potential inhibitors for 12 targets from the (S)-adenosyl-L-methionine-dependent methyltransferase (SAM MTase) family. In total, 1740 molecules were collected from experimental data and from the DUD-E website (446 actives and 1294 decoys), and docked using Glide. An MLP was chosen and the complexes encoded by the TIFP. The data set was randomly split into training and test sets with a 10:1 ratio. In a binary classification experiment, the MLP showed an AUC of 0.86 and an $EF_{5\%}$ of 3.46 on the test set, and thus, outperformed the traditional docking tools Glide (0.75 and 2.97), and AutoDock vina (0.61 and 0.99).

3D Grid-Based Studies

Many methods using a 3D grid representation of a protein-ligand complex—comparable to pixels in 3D images—for affinity prediction, have evolved over the last years [88,89,92,93], especially due to the increased popularity of deep CNNs.

One of the first published models, AtomNet [88] uses a CNN, composed of an input layer, i.e., the vectorized 3D grids, several 3D convolutional and fully-connected layers, as well as an output layer, which assigns the probability of the two classes: active and inactive. Among other data sets, the DUD-E benchmark, consisting of 102 targets, over 20,000 actives and 50 property matched decoys per active compound, was used for evaluation. 72 targets were randomly assigned as training set, the remaining 30 targets as test set (DUDE-30). For each target, a holo structure from the scPDB [143] is used to place the grid around the binding site and multiple poses per molecule are sampled. Finally, the grid is fixed to a side length of 20 Å and a 1 Å grid spacing, in which each grid point holds some structural feature such as atom-type or IFP. On the DUDE-30 test set, AtomNet achieves a mean AUC of 0.855 over the 30 targets, thus outperforming the classical docking tool smina [144] (mean AUC of 0.7). Furthermore, AUC values greater than 0.9 were reported for 46% of the targets in the DUDE-30 test set.

Similarly, BindScope [93] voxelizes the binding pocket by a 16 Å grid of 1 Å resolution, molecules are placed using smina, and each voxel is assigned a distance-dependent input based on eight pharmacophoric feature types [91]. The 3D-CNN model architecture was adapted from DenseNet [145] and yields a mean AUC of 0.885 on the DUD-E benchmark in a five-fold cross-validation (folds were assigned based on protein sequence similarity-based clusters). Comparable AUC values on the DUD-E set were reported by Ragoza et al. [146] (mean AUC of 0.867), a similar grid-based CNN method, which outperformed AutoDock vina on 90% of the targets.

DeepAtom [92] uses a 32 Å box with 1 Å resolution and assigns a total of 24 features to each voxel (11 Arpeggio atom types [147] and an exclusion volume for ligand and protein respectively) in individual channels to encode the protein-ligand complex. The PDBbind v.2016 served as baseline benchmark data, split into 290 complexes for testing and 3767 non-overlapping complexes between the refined and core sets for training and validation. In particular, each original example gets randomly translated and rotated for data argumentation, which aims to improve the learning capacity. The performance of the built 3D-CNN model, trained on the PDBbind refined set, in predicting the affinity for the core set in a regression setting was reported with a low mean RMSE of 1.318 (*R* of 0.807) over five runs. In this case, DeepAtom outperformed RF-Score [148], a classical ML method (mean RMSE of 1.403), as well as Pafnucy [89] (mean RMSE of 1.553), a similar 3D-CNN method, trained and applied to the same data using their open-source code. Note that in the original publication, Pafnucy [89] achieved prediction results with an RMSE of 1.42 on the PDBbind core set v.2016. In a further study, the training set for DeepAtom was extended by combining BindingMOAD and PDBbind subsets, resulting in 10,383 complexes. While the mean RMSE of DeepAtom slightly decreased to 1.232, the *R* value increased to 0.831 for the PDBbind core set.

The presented examples show the effectiveness of 3D grid-based encodings and CNN models for affinity prediction, which seem to be well suited to implicitly capture the variety of information important for ligand-binding. However, disadvantages are the high memory demand of 3D grids and CNNs, as well as the implicit grid boundary definition to capture the protein-ligand interactions.

Graph-Based Studies

Graph neural networks have proven to be some of the most effective deep learning models, easily reaching state-of-the-art performance. In this context, two recent applications of such models in virtual screening are described.

Lim et al. [95] construct a graph representation of the protein predicted binding pose complex, obtained using smina [144] and train a graph neural network to successfully predict activity. The node feature vector concatenates atomic information from the ligand and from the protein. The features considered for both are the one-hot encoding of the following atomic properties: type (10 symbols), degree (6 possibilities for 0 to 5 neighbors), number of hydrogens (5 entries for 0 to 4 possible attached Hs), implicit valence of electrons (6 entries) and a binary entry for aromaticity. This leads to 28 entries for the ligand, another 28 for the protein, generating a feature vector of size 56. The 3D information is encoded in the two matrices $A^{1}$ and $A^{2}$ described in Section 2.1.3, for covalent and non-covalent interactions, respectively. The model applies four layers of GAT (gate augmented graph attention) to both $A^{1}$ and $A^{2}$, before aggregating the node information using summation. A 128-unit fully-connected layer is then applied to this vector, which leads to binary activity prediction. DUD-E is used for training and testing the VS performance, where the training set contains 72 proteins with 15,864 actives and 973,260 inactives and the test set another 25 proteins with 5841 actives and 364,149 inactives. The AUC value on the test data set reaches 0.968, which is high compared to the value of 0.689 obtained with the smina docking tool. The model also obtains better scores than other deep learning (DL) models such as the CNN-based models AtomNet [88] and the one developed by Ragoza et al. [146]. The same trend holds for the reported PDBbind data set study. However, when testing their model and docking results on external data sets such as ChEMBL and MUV, the performance drops, hinting to the fact that the DL model might not be able to generalize to the whole chemical space.

The graph convolution family PotentialNet developed by Feinberg et al. [96] predicts protein-ligand binding at state-of-the-art scales. The atomic features are atom type, formal charge, hybridization, aromaticity, and the total numbers of bonds, hydrogens (total and implicit), and radical electrons. The structure between the atoms is described using $A\in R^{N\times N\times N_{et}}$, the extended representation of an adjacency matrix, as described in Section 2.1.3. The PotentialNet model uses a Gated Graph Neural Network (GGNN), which means that unlike GNNs, the update function is a GRU, leading to the new node vector, depending on its previous state and the message from its neighbors, in a learned manner. PotentialNet also considers different stages, where stage 1 makes use of only the bonded part of the adjacency matrix, leading to node updates for connectivity information, stage 2 considers spatial information, and stage 3 sums all node vectors from ligands before applying a fully-connected layer for binding affinity prediction. The model is trained on complexes of the PDBbind v.2007 using a subset of size 1095 of the initial refined set for training, and then tested on the core set of 195 data points. The model reaches a test $R^{2}$ value of 0.668 and a test *R* value of 0.822, outperforming RF-Score (*R* of 0.783) and X-Score (*R* of 0.643) ([96], Table 1). However, similar results were reported by the CNN-based model TopologyNet [97], introduced below.

Other Studies

In MathDL [139] and TopologyNet [97], the complexes—and thus the interactions between protein and ligand—are encoded using methods from algebraic topology. In MathDL, advanced mathematical techniques (including geometry, topology and/or graph theory) are used to encode the physicochemical interactions into lower-dimensional rotational and translational invariant representations. Several CNNs and GANs (Generative Adversarial Networks) are trained on the PDBbind v.2018 data set and applied on the data of the D3R Grand Challenge 4 (GC4), a community-wide blind challenge for compound pose and binding affinity prediction [149]. The models are among the top performing methods in pose prediction on the beta secretase 1 (BACE) data set with an RMSD [126] of 0.55 Å and a high ρ of 0.73 in affinity ranking of 460 Cathepsin S (CatS) compounds. Additionally good performance was reported on the free energy set of 39 CatS compounds. TopologyNet [97], a family of multi-channel topological CNNs, represent the protein-ligand complex geometry by a 1D topological invariant (using element-specific persistent homology) for affinity prediction and protein mutation. In the affinity study, a TopologyNet model (TNet-BP) is trained on the PDBbind v.2007 refined set (excluding the core set) and achieves an *R* of 0.826 and an RMSE of 1.37 in $pK_{d}$/$pK_{i}$ units. The $pK_{d}$ and $pK_{i}$ values describe the negative decimal logarithm of $K_{d}$ and $K_{i}$ values, respectively. Thus, TNet-BP seems to outperform other well-known tools such as AutoDock vina and GlideScore-XP on this data set (note that the results are adopted from the original study by Li et al. [150]).

DeepBindRG [151] and DeepVS [99] focus on the interacting atom environments in the complex using atom pair and atom context encodings, respectively. DeepBindRG, a CNN model trained on PDBbind v.2018 (excluding targets that appear in the respective test set), achieves good performance on independent data sets such as the CASF-2013 and DUD-E subsets, with an RMSE varying between 1.6 and 1.8 for a given protein and an *R* between 0.5 and 0.6. With these values, DeepBindGP performs slightly better than AutoDock vina, while being in a similar range as Pafnucy [89]. DeepVS, another CNN model, trained and tested on the DUD data set using leave-one-out cross-validation outperforms, with an AUC of 0.81, AutoDock vina 1.2 which has an AUC value of 0.62.

### 3.2. Pair-Based Models

In this section, pair-based/PCM models from the literature are presented (see Table 4). As discussed above, pair-based methods do not require the crystal structure of a protein, nor the docked pose of a ligand. Indeed, the ligand is modeled independently from the protein and vice versa. This framework resembles proteochemometric (PCM) models [22,23], whereas the cross-term, including some interactions between the ligand and the protein, which can be used in PCM, is not present in the herein reported pair-based setting. The discussed studies are grouped by the type of ligand encoding they use: SMILES, fingerprint and graph.

Ligand as SMILES

In 2018, Öztürk et al. [60] proposed the DeepDTA (Deep Drug-Target Binding Affinity Prediction) regression model which takes the SMILES and a fixed length truncation of the full protein sequence as features for the ligand and protein, respectively. In the study, two kinase-focused data sets are used: the Davis data [119] and the KIBA data [120] with roughly 30,000 and 250,000 data points, respectively. The first reports $K_{d}$ values, which represents the dissociation constant, while the second reports Kinase Inhibitor BioActivity (KIBA) scores, which combines information from $IC_{50}$, $K_{i}$ and $K_{d}$ measurements. As input for the CNN, both the SMILES and the protein sequence are label encoded independently. The authors apply convolutions to the embeddings of each object, before concatenating them and predicting the $pK_{d}$ value or KIBA score, depending on the data set used. The data are randomly split into six equal parts where one of them is used as a test set to evaluate the model and the five remaining compose the folds for cross-validation and parameter tuning. On the Davis and KIBA test sets, the model exhibits an MSE of 0.261 and 0.194, respectively (see [60], Tables 3 and 4), which outperforms baselines such as KronRLS [157], a variation of least squares regression and SimBoost [158], a tree-based gradient boosting method. The success of the deep learning model could be explained by the use of convolution layers which are able to extract information from the protein-ligand pair.

The same authors extended DeepDTA to WideDTA [152]. This time, instead of only considering the SMILES label encoding for the ligand, substructure information is also included where a list of the 100,000 most frequent maximum common substructures defined by Woźniak et al. [159] are used. For the protein description, approximately 500 motifs and domains are extracted from the PROSITE database [160] and label encoded. The deep learning architecture is similar to DeepDTA, but WideDTA does achieve slightly better results, for example an MSE of 0.179 on the KIBA data ([152], Table 5).

In another study, Karimi et al. [74] use SMILES as ligand and structural property sequences as protein descriptors to predict protein-ligand affinities. The first learning task is an auto-encoder which aims at giving a latent representation of a compound-target pair. Once the neural network is trained in an unsupervised setting, the resulting fingerprint is then fed to recurrent plus convolution layers with an attention mechanism to predict $pIC_{50}$ values. The BindingDB data set [161], containing close to 500,000 labeled protein-compound pairs after curation, is used for their study. After removing four protein classes as generalization sets, the remaining ∼370,000 pairs are split into train (70%) and test (30%) sets. On the test set, while RF yields an RMSE of 0.91 and a Pearson’s *R* of 0.78, the DeepAffinity model reaches an *R* of 0.86 and a lower RMSE of 0.73 ([74], Table 2), thus outperforming conventional methods such as RF. A GCNN is also tested on the graph encoding of the compounds, but this alternative did not show improvements with respect to the SMILES notation.

Ligand as Fingerprint

The DL-CPI model suggested by Tian et al. [153] stands for Deep Learning for Compound-Protein Interactions and applies four fully-connected hidden layers to a 6404 long input binary vector which is the concatenation of compound and protein features; 881 entries for substructure identification in the ligand and another 5523 for Pfam [73] identified protein domains. Using five-fold cross-validation, the AUC varies between 0.893 and 0.919 depending on the ratio of negative samples in the data set for DL-CPI and between 0.687 and 0.724 for an RF model ([153], Table 2). The high accuracy performance is explained by the abstraction coming from the hidden layers of the network.

The study by Kundu et al. [65] compares various types of models trained and tested on a subset of 2864 instances of the PDBbind v.2015 data set. The 127 long input feature vector combines features of the protein, such as the percentage of amino acids, the accessible surface area of the protein, the number of chains, etc., as well as physicochemical (e.g., molecular weight, topological surface area, etc.) and structural properties (e.g., ring count) of the ligand. RF is shown to outperform models such as MLP and SVM in the task of predicting inhibition constant ($K_{i}$) and dissociation constant ($K_{d}$) values. One possible reason for these results might originate from the size of the data set: RF models can be very successful when the available data is small.

The study undertaken by Sorgenfrei et al. [70] focuses on the RF algorithm. They encode the ligand with Morgan fingerprints and note that using z-scales descriptors from the binding site of the protein highly improves the performance of the model compared to the baseline which only considers the one-hot encoded ID of the target. The data set used contains over 1,300,000 compound-kinase activities and comes from combined sources such as ChEMBL and the KIBA data provided by Tang et al. [120]. The activity threshold for $pIC_{50}$ values ($pIC_{50}=-log_{10}\left(IC_{50}\right)$) is set at 6.3. On a test set in which both target and compound are left out during training, the AUC reaches a value of 0.75 ([70], Table 1), justifying the usefulness of pair-based/PCM methods in hit identification.

Morgan fingerprints are also used in the study by Lee et al. [154] to represent the ligand, while the full raw protein sequence is used as protein input. The deep learning model, DeepConv-DTI, consists of convolutions applied to the embeddings of the full protein sequence (padded to reach the length of 2500), which are then combined with the ligand descriptors in fully-connected layers to predict whether the drug is a hit or not. The model is built on combined data from various open sources, such as DrugBank [162], KEGG [163] and IUPHAR [164]. After curation and generating negative samples, the training set contains close to 100,000 data points. The model is externally tested on PubChem where both protein and compound had not been seen during training. DeepConv-DTI reaches an accuracy close to 0.8 ([154], Figure 3D) and seems to outperform the DeepDTA model by Öztürk et al. [60], see ([154], Figure 4).

Ligand as Graph

In the study by Torng and Altman [57], GCNNs are used on both target and ligand. The residues in the binding pocket of the protein correspond to the nodes and the 480 long feature vector, computed with the program developed by Bagley and Altman [165], represents their physicochemical properties. The small molecule is also treated as a graph and properties such as the one-hot encoded atomic element, degree, attached hydrogen(s), valence(s), and aromaticity, are included in the 62 long feature vector. Graph convolutional layers are applied to both graphs independently and the resulting vectors from both entities are concatenated. A fully-connected layer is applied to the concatenated vector to learn the interaction between the molecule and the target, leading to an interaction vector which is then used to predict binding or non-binding. The model is trained on the DUD-E data set and externally tested on MUV, and reaches an AUC value of 0.621, which is better than results from 3D CNNs, AutoDock vina and RF-Score ([57], Table 2).

The research undertaken by the authors Jiang et al. [155] uses a similar workflow, where GNNs are applied to both the ligand graph and the protein graph based on the contact map. More precisely, the atomic properties of the ligand nodes are the one-hot encoding of the element (44 entries), the degree (11 entries), the total (implicit and explicit) number of attached hydrogens (11 entries), the number of implicit attached hydrogens only (11 entries), and aromaticity (binary value), leading to a vector of length 78. The atomic properties of the protein include the one-hot encoding of the residue (21 entries), the position-specific scoring matrix for amino acid substitutions (21 entries), and the binary values of the 12 following properties: being aliphatic, aromatic, polar, acidic, or basic, the weight, three different dissociation constants, the pH value and hydrophobicity at two different pH values. The contact map, which can be computed using the PconsC4 tool [166] directly from the protein sequence, can be used as a proxy for the adjacency matrix. Given the graph representation for the ligand as well as the protein, three graph convolutional, pooling layer, and fully-connected layers are subsequently applied to both ligand and protein independently, then concatenated, and finally the binding affinity is predicted after another two fully-connected layers. The deep learning model is called DGraphDTA, which stands for “Double Graph Drug–Target Affinity predictor”. The MSE on the Davis and KIBA data sets are as low as 0.202 and 0.126, respectively and DGraphDTA seems to give better results than both DeepDTA and WideDTA, see ([155], Tables 7 and 8). This hints to the fact that graph representations are well suited for drug-target interaction prediction.

The PADME model, an acronym for “Protein And Drug Molecule interaction prEdiction” [156], suggests two variants for ligand encoding in the regression context of drug-target interaction prediction. The ECFP fingerprint (implemented as the Morgan fingerprint in the code) as well as the graph encoding are used along side a 8421 long protein feature vector describing the sequence composition (8420 entries for the amino acid, dipeptide, and tripeptide composition computed using the propy tool [167]) and one entry for phosphorylation. The deep learning model is adapted depending on the encoding of the ligand. In the case of circular fingerprint, the protein and ligand vectors are concatenated to form a “combined input vector”, on which fully-connected layers are then applied. In the graph setting, a graph layer is applied to the ligand, resulting in a vector, which is then again concatenated to the protein vector as in the previous case. The regression models (either graph or circular fingerprint) consistently outperform baseline models such as KronRLS and SimBoost. The simulations are run on several kinase data sets such as Davis [119] and KIBA [120]. Using cross-validation schemes that involve testing the model on the fold for which no protein was trained on, the RMSE on the KIBA data with the PADME graph setting is 0.6225 and on the Davis data with the circular fingerprint setting is 0.5639 ([156], Table 2). This study provides further evidence that deep learning models could indeed improve drug-target prediction compared to standard machine learning algorithms.

## 4. Conclusions and Discussion

Over the last decade(s), a wave of deep learning methods and applications to boost virtual screening, i.e., affinity—but also other properties, such as ADMETox—prediction, has emerged. This development is coupled to the availability of more and more compounds, structures and mapped bioactivity data, together with novel encoding techniques and deep learning technologies. These include not only model architectures, that seem to fit well the nature of biological objects such as ligands and proteins, but also open-source software and computer hardware evolution.

Around thirty papers related to deep learning-based virtual screening are described in details in this review (see Table 3 and Table 4). Most of these studies were published between 2018 and 2020, giving an overview of the current state-of-the-art and the advancements of deep learning in the field. The encodings for protein and ligand (Section 2.1), the machine learning models (Section 2.2), the data sets (Section 2.3) as well as the model performances (Section 3) are reported and put in context. These studies show overall very promising results on typical benchmarks and often outperform the respective classical approach chosen for comparison, such as docking or more standard machine learning models. This is also exemplified on the Merck Molecular Activity Kaggle competition data, where deep neural networks have shown to routinely perform better than random forest models [168]. Similarly, in other blind challenges for pose and affinity prediction such as the D3R grand challenges, deep learning-based methods increasingly make it to the top ranges ([149], Table 1). One possible reason for such outstanding achievements may be explained by the way biological entities are encoded: for example, rather than using human-engineered descriptors, features are learned by the models. Also, novel encodings, such as voxels (where physicochemical atomic properties are pinned to locations in 3D space) and graphs (that describe the connectivity, bonded and non-bonded, between the atoms), seem to capture well the variety of information important for ligand-binding. For example, DeepAtom [92], a 3D grid-based method where each grid cell is assigned a different physicochemical property seems well suited to model the complexity of protein-ligand binding using 3D information. Encoding chemical and biological objects in graph form also seems to be very fitting, as shown in the study by Lim et al. [95] and the DGraphDTA model by Jiang et al. [155].

Nevertheless, several challenges still remain open and new ones have also emerged, including: 1. precision of chemical encoding, 2. generalization of chemical space, 3. lack of (big and high-quality) data, 4. comparability of models, and 5. interpretability. All of which will be discussed in the following.

1. *Precision of chemical encoding:* The better performance of structure-based methods using ML-based vs. classical SFs is often attributed to the avoidance of a pre-determined functional form of the protein-ligand complexes, meaning that the precision of the chemical description does not necessarily lead to more accurate binding affinity prediction [169]. Contributing factors might be associated with: 1. modeling assumptions, where more precise descriptions may introduce errors. 2. The dependence of encoding and regression technique: more precise description might produce longer and sparser features which could be problematic in cases such as RF models. 3. Restrictions to data in the bound state, neglecting contribution from both partners in solvation and induced fit phenomena. Or missing consideration of conformational heterogeneity, where multiple conformations might co-exist with different probabilities.

2. *Generalization of chemical space:* As mentioned in the work by Lim et al. [95], although some deep learning models perform outstandingly well, there seems to still exist some issues exploring the whole chemical space, a challenge also occurring in classical machine learning methods. Some less successful results have been detected when evaluating some models on external data sets, showing that since the data used for training is not representative of the immense chemical space, the model, instead of learning and exploring it, is rather memorizing patterns from it [170].

3. *Lack of (big and high-quality) data:* Deep learning is very data greedy and usually, the bigger the training set is, the better the results. Goodfellow et al. [32] suggest that a model trained on a data set of size of the order of 10 million may surpass human performance. However, as previously discussed, biochemical data are still considerably smaller than, for example, image or video data sets. Therefore, depending on the data at hand, choosing more standard machine learning approaches, or more shallow neural networks, that require less parameter training, may perform just as well. Examples are shown in the studies by Kundu et al. [65], which employs random forest for activity prediction, or by Göller et al. [171], which summarizes DL and ML models for ADMETox predictions. Another alternative is to find a way to acquire more data, through, for example, data augmentation. In image classification, this can be done using image rotating, cropping, recoloring, etc., which can be adapted to virtual screening tasks. The Pharm-IF method [84] performs better with more crystal structures or by employing additional docking poses. DeepAtom [92] translates and rotates the protein-ligand complex to gain more training data. In QSAR predictions, using SMILES augmentation has also become popular as means to enlarge the training set [59,61]. Note that not only the quantity of data, but also its quality is often unsatisfactory, such as low resolution of crystal structures or relying on docked poses, as well as activity data taken from various experiments (and conditions) providing different measurements, e.g., $K_{d}$, $K_{i}$, $IC_{50}$ or $EC_{50}$ (which is the measured half maximal effective concentration of a drug).

4. *Comparability of models, benchmark data, open-source:* Reviewing a multitude of studies and wanting to compare and rank them is understandable, but also unreasonable for several reasons, starting with the data and the splits. Models that have been trained on different data or even different tasks should hardly be compared; a regression task or a classification task, even when using similar performance metrics, are not analogous. Assuming that the models do use the same data, if the splits are different, then the evaluation can no longer be directly compared. Assuming now that the splits are identical, then if the metrics used are different, again no fair comparison can be made, as pointed out by Feinberg et al. [96]. This means that there are a chain of elements that have be considered before comparing and ranking methods blindly. To this end, two major elements become crucial: 1. open-source data and 2. open-source code.

Having benchmark data sets freely available—together with a code basis—such as MoleculeNet [25], TDC [133] or work by Riniker and Landrum [134], and updated regularly, such as ChEMBL, is highly beneficial for academic research and method publication. Moreover, having access to the source code of newly developed methods and being able to reproduce results is also becoming more and more essential in the field especially as the number of models developed is becoming larger (as embraced by the FAIR principles [172]).

Moreover, while several data sets are available to benchmark the performance of different approaches in VS, Sieg et al. [121] recently elaborated on the need of bias control for ML-based virtual screening studies. Several types of biases exist. For example, domain bias, which maybe be due to insufficient generalization as discussed above, but still acceptable, if the models are applied in a narrow chemical space. Nevertheless, non-causal bias is dangerous, when there is correlation but no causation. While mainly focusing on structure-based ML models for VS on DUD, DUD-E and MUV, Sieg et al. [121] found that small molecule features dominated the predictions across dissimilar proteins even when structure-based methods/descriptors are used. Thus, special care needs to be taken when methods and descriptors are evaluated on benchmark sets, if the compilation protocol of the benchmark is suited for the context of the methodology. In another study, Chen et al. [173] also claim hidden analogue and decoy bias in the DUD-E database that may lead to superior performance of CNN models during VS. Thus, there is urgent need for bias control in benchmarking data sets, especially for structure-enabled ML-based VS.

5. *Interpretability:* With the rise of deep learning, the complexity of the architectures and the depth of the models comes the issue of interpretability. Such models are often considered as black boxes and understanding the mechanism in the hidden layers is a challenge. However, research undertaken in this direction aims at deciphering what the algorithm has learned [101,174]. This may also be important in detecting bias in the data [121].

For further considerations on the type, quality and quantity of the data as well as the challenges of DL models built thereof to impact different areas of drug discovery, the reader is kindly referred to two recent reviews by Bender and Cortés-Ciriano [175,176].

In this work, the recent progress in DL-based VS methods has been reviewed, exemplifying the boost in development and application over the last few years. While some challenges due to, for example data coverage and unbiased evaluation sets, molecular encoding and modeling the respective biological protein-ligand binding event still remain, the reported results show the unprecedented advances in the field.

## Figures and Tables

**Table 1:** Structure and bioactivity data sets. The table lists common labeled data sets used in virtual screening studies. Freely available data is increasing each year and is an essential element for affinity prediction using machine and deep learning models. The table summarizes the name, the size and the content covered as well as links to the respective website.

Name	Size and Content ^1^	Availability ^2^
PDBbind v.2019	structures + activities: general: 21,382; refined: 4852; core: 285	http://www.pdbbind.org.cn
BindingDB	2823 structures + activities 2,229,892 activities	https://www.bindingdb.org
BindingMOAD 2019	38,702 structures 15,964 structures + activities	https://bindingmoad.org
PubChem BioAssay 2020	>280 million activities	https://pubchem.ncbi.nlm.nih.gov
ChEMBL v.28	17,276,334 activities	https://www.ebi.ac.uk/chembl

^1^ Structures refers to protein-ligand X-ray structures. Activities refer to measured compound-target bioactivity data points as reported in the respective data source. ^2^ Websites accessed on 18 March 2021.

**Table 2:** Benchmark data sets. Evaluating novel models on labeled benchmark data is crucial for any machine learning task, including deep learning-based virtual screening. The table depicts some commonly used databases with their respective size, the origin of the data, provided information (affinity or activity) as well as their availability through websites (accessed on 18 March 2021).

Name	Size	Data Source	Label	Availability
CASF-2016	57 targets 285 complexes	PDBbind	affinitiy	http://www.pdbbind.org.cn/casf.php
DUD-E	102 targets 22,886 actives 50 decoys per active	PubChem, ZINC	active/decoy	http://dude.docking.org
MUV	17 targets ∼90,000 compounds	PubChem, ZINC	active/decoy	MUV@TU Braunschweig ^1^

^1^ Website: https://www.tu-braunschweig.de/pharmchem/forschung/baumann/muv.

**Table 3:** Complex-based models. Summary of recent work using a protein-ligand complex for active molecule or binding affinity prediction. The year of publication, the name of the authors or the model, the complex encoding and the machine/deep learning model(s) are shown in the respective columns. Classification (class.) implies predicting e.g. hit or non-hit, whereas regression (reg.) evaluates e.g., $pIC_{50}$ values. CNNs, coupled with 3D grids, have become frequent in state-of-the-art studies.

Year	Name	Complex Encoding ^1^	ML/DL Model	Framework
2010	Sato et al. [84]	IFP	SVM, RF, MLP	class.
2016	Wang et al. [135]	IFP	Adaboost-SVM	class.
2019	Li et al. [136]	IFP	MLP	class.
2018	gnina [90]	3D grid	CNN	class.
2018	KDEEP [91]	3D grid	CNN	reg.
2018	Pafnucy [89]	3D grid	CNN	reg.
2018	DenseFS [137]	3D grid	CNN	class.
2019	DeepAtom [92]	3D grid	CNN	reg.
2019	Sato et al. [138]	3D grid	CNN	class.
2019	Erdas-Cicek et al. [94]	3D grid	CNN	reg.
2019	BindScope [93]	3D grid	CNN	class.
2018	PotentialNet [96]	graph	GGNN	reg.
2019	Lim et al. [95]	graph	GANN	class.
2017	TopologyNet [97]	topol.	CNN	reg.
2019	Math-DL [139]	topol.	GAN, CNN	reg.
2018	Cang et al. [140]	topol.	CNN	reg.
2016	DeepVS [99]	atom contexts	CNN	class.
2019	OnionNet [141]	atom pairs	CNN	reg.
2020	Zhu et al. [98]	atom pairs	MLP	reg.

^1^ Abbreviations: IFP: interaction fingerprints, topol.: algebraic topology.

**Table 4:** Pair-based models. The listed models consider information from the protein and the ligand, but the encodings are built independently of each other. The year of publication, the name of the authors or the model, the ligand and the protein encodings and the machine/deep learning model(s) are shown in the respective columns. Classification (class.) implies hit or non-hit, whereas regression (reg.) evaluates an affinity measure, for example $pIC_{50}$ values. Graphs and associated GCNNs have become prominent in recent years.

Year	Name	Ligand Encoding ^1^	Protein Encoding ^1^	ML/DL Model	Framework
2018	DeepDTA [60]	SMILES	full seq.	CNN	reg.
2019	WideDTA [152]	SMILES & MCS	full seq. & domains/motifs	CNN	reg.
2019	DeepAffinity [74]	SMILES	struct. property seq.	RNN+CNN	reg.
2016	DL-CPI [153]	substructure FP	domains	MLP	class.
2018	Kundu et al. [65]	div. feat. FP	div. feat. FP	RF & SVM & MLP	reg.
2018	Sorgenfrei et al. [70]	Morgan FP	z-scales	RF	class.
2019	DeepConv-DTI [154]	Morgan FP	full seq.	CNN	class.
2019	Torng and Altman [57]	graph	graph	GCNN	class.
2020	DGraphDTA [155]	graph	graph	GCNN	reg.
2018	PADME [156]	graph (or Morgan FP)	seq. comp.	GCNN (or MLP)	reg.

^1^ Abbreviations: FP: fingerprint, MCS: maximum common substructure, div. feat.: diverse feature count, physicochemical and structural properties, seq.: sequence, comp.: composition.

## Data Availability

The Python code to generate most components of the figures in the review is available on GitHub at https://github.com/volkamerlab/DL_in_VS_review, using packages such as RDKit [63], NGLview [177], the Open Drug Discovery Toolkit (ODDT) [178] and PyMOL [179].

## Abbreviations

The following abbreviations are used in this manuscript:

**General**	
HTS	High-throughput screening
VS	Virtual screening
SF	Scoring function
QSAR	Quantitative structure–activity relationship
PCM	Proteochemometric
FDA	Food and Drug Administration

**Machine learning**	
ML	Machine learning
DL	Deep learning
SVM	Support vector machine
RF	Random forest
NN	Neural network
ANN	Artificial neural network
MLP	Multilayer perceptron
CNN	Convolutional neural network
RNN	Recurrent neural network
GNN	Graph neural network
GCNN	Graph convolution neural network
GRU	Gated recurrent unit
GGNN	Gated graph neural network
GANN	Graph attention neural network
GAN	Generative adversarial network

**Encoding**	
SMILES	Simplified molecular input line entry system
ECFP	Extended-connectivity fingerprint
MCS	Maximum common substructure
IPF	Interaction fingerprint
ID	Identifier

**Metrics**	
MSE	Mean squared error
RMSE	Root mean squared error
RMSD	Root mean square deviation
ROC	Receiver operating characteristic
AUC	Area under the ROC curve
EF	Enrichment factor

**Data**	
PDB	Protein Data Bank
KIBA	Kinase inhibitor bioactivity
MUV	Maximum unbiased validation
TDC	Therapeutics Data Commons
DUD	Directory of useful decoys
CASF	Comparative assessment of scoring functions

## Appendix B. Evaluation Strategies and Metrics

In the following section, metrics commonly used to evaluate the performance of a given model are described. Depending on the learning task, these metrics may vary. First, metrics used in binary prediction leading to a classification framework are discussed. Then, the typical metrics used in the regression framework are described and finally the metrics that can be applied in both cases.

### Appendix B.1. Classification

Often times, the area under the ROC curve (AUC) [180] is reported, when the learning task requires determining if there is a hit or non-hit given a ligand and a protein. The receiver operating characteristic (ROC) curve plots the true positive rate (*y*-axis) versus the false positive rate (*x*-axis) when the threshold of the classifier varies. Obtaining a 100% true positive rate and 0% false positive rate would be the ideal setting and would yield an AUC value of 1. As a measure to compare models, the closer the AUC value is to 1, the better the classifier. A detailed explanation of ROC curves and AUC values can be found in [181].

The accuracy (Acc) [182] is defined by

$$\mathrm{Acc}=\frac{\mathrm{TP}+\mathrm{TN}}{\mathrm{TP}+\mathrm{TN}+\mathrm{FN}+\mathrm{FP}},$$

where TP, TN, FN, FP are the true positives, true negatives, false negatives and false positives, respectively.

The enrichment factor (EF) [127] is a measure for evaluating screening efficiency. At a pre-defined sampling percentage χ, ${\mathrm{EF}}_{\chi \%}$ shows the proportion of true active compounds in the sampling set in relation to the proportion of true active compounds in the whole data set and is defined by

$${\mathrm{EF}}_{\chi \%}=\frac{\frac{n_{s}}{N_{s}}}{\frac{n}{N}},$$

where *N* is the number of compounds in the entire data set, *n* the number of compounds in the sampling set, $N_{s}$ the number of true active compounds in the entire data set, and $n_{s}$ the number of true active compounds in the sampling set.

### Appendix B.2. Regression

In the following, $y_{i}$ is assumed to be the *i*th true value of *y*, $\hat{y_{i}}$ the *i*th predicted value of $\hat{y}$ by the algorithm, and *n* the number of data points considered.

The mean squared error (MSE) measures the difference between *y* and $\hat{y}$ using the Euclidean norm:

$$\mathrm{MSE}=\mathrm{MSE}\left(y,\hat{y}\right)=\frac{1}{n}\sum_{i=1}^{n}{\left(y_{i}-\hat{y_{i}}\right)}^{2}.$$

The root mean squared error (RMSE), as its name suggests, is simply the squared root of the MSE,

$$\mathrm{RMSE}=\sqrt{\mathrm{MSE}}.$$

Since the MSE and the RMSE are metrics that represent the difference between the true and predicted values, the smaller these errors are, the better the model.

### Appendix B.3. Classification & Regression

$R^{2}$ is a measure of goodness of fit which can be applied in both the classification and the regression framework [183,184] and is defined by

$$R^{2}=1-\frac{\sum_{i}{\left(y_{i}-\hat{y_{i}}\right)}^{2}}{\sum_{i}{\left(y_{i}-\bar{y}\right)}^{2}},$$

where

$$\bar{y}=\frac{1}{n}\sum_{i=1}^{n}y_{i}.$$

The closer is the $R^{2}$ to 1, the better the fit. $R^{2}=0$ when the model predicts all values to the mean $\bar{y}$.

The Pearson’s correlation coefficient *R* [123,155,185] defined below is also often used.

$$R=\frac{\sum_{i}\left(y_{i}-\bar{y}\right)\left(\hat{y_{i}}-\bar{\hat{y}}\right)}{\sqrt{\sum_{i}{\left(y_{i}-\bar{y}\right)}^{2}\sum_{i}{\left(\hat{y_{i}}-\bar{\hat{y}}\right)}^{2}}}.$$

The Spearman’s rank correlation coefficient ρ [124,125] can be calculated with

$$\rho =1-\frac{6\times \sum_{i}{\left(\mathrm{rank}\left(y_{i}\right)-\mathrm{rank}\left(\hat{y_{i}}\right)\right)}^{2}}{n\times (n^{2}-1)},$$

where *n* is the number of observations. In contrast to Pearson’s correlation coefficient *R*, the ranks between the observed ($y_{i}$) and predicted (${\hat{y}}_{i}$) values are compared.
